# Piperlongumine Induces Apoptosis and Synergizes with Doxorubicin by Inhibiting the JAK2-STAT3 Pathway in Triple-Negative Breast Cancer

**DOI:** 10.3390/molecules24122338

**Published:** 2019-06-25

**Authors:** Di Chen, Yangmin Ma, Peiqi Li, Meng Liu, Yuan Fang, Jiejie Zhang, Bilin Zhang, Yuyu Hui, Yue Yin

**Affiliations:** 1 Shaanxi Key Laboratory of Chemical Additives for Industry, Shaanxi University of Science and Technology, Xi’an 710021, China; observercd@163.com (D.C.); 18392995152@163.com (Y.H.); 2 School of Food and Biological Engineering, Shaanxi University of Science and Technology, Xi’an 710021, China; 3 Institute of Basic Medical Sciences, Xi’an Medical University, Xi’an 710021, China; L316844007@gmail.com (P.L.); 13468606138@163.com (M.L.); FY15802957566@gmail.com (Y.F.); 18089100363@163.com (J.Z.); z17795781759@163.com (B.Z.); 4 School of Public Health, Xi’an Jiaotong University Health Science Center, Xi’an 710061, China; evelynhouse@xjtu.edu.cn

**Keywords:** triple-negative breast cancer, piperlongumine, doxorubicin, apoptosis, JAK2, STAT3

## Abstract

Triple-negative breast cancer (TNBC) lacks major effective target molecules and chemotherapy remains the current main treatment. However, traditional chemotherapy drugs, such as doxorubicin (DOX), cause serious side effects and have a poor prognosis. Piperlongumine (PL), a natural alkaloid, has showed selective anticancer effects and is expected to become a new strategy against TNBC. In our research, cell viability, colony formation, flow cytometry, Western blot, and tumor xenograft model assays were established to evaluate the suppression effect of PL and DOX alone and in combination. Data showed that PL could effectively inhibit cell growth and induce apoptosis in two TNBC cell lines. We also demonstrated for the first time that the combination treatment of PL and DOX synergistically inhibited cell growth and induced apoptosis in TNBC cells. The suppression of STAT3 activation was indicated to be a mechanism of the anticancer effect. Moreover, the effectiveness of this combination was confirmed in a tumor xenograft model. These results revealed that inhibition of the JAK2-STAT3 pathway was a key anticancer mechanism when treated with PL alone or combined with DOX, suggesting that the combination of PL and chemotherapy drugs may be a potential strategy for the clinical treatment of TNBC.

## 1. Introduction

Triple-negative breast cancer (TNBC) has a worse overall survival than non-TNBC [1]. Currently, approved drugs are mainly targeted at human epidermal growth factor receptor-2-positive breast cancer, and there is less effective treatment for TNBC due to the lack of major effective target molecules [2]. Patients with TNBC are forced to receive cytotoxic chemotherapy, suffering from toxic side effects and having a poor prognosis [3]. Therefore, a new strategy for TNBC treatment is urgently needed.

Current clinical treatment options for breast cancer include surgery, radiotherapy, chemotherapy, and combinations thereof, with chemotherapy playing a crucial role. As one of the representative drugs of anthracyclines, doxorubicin (DOX) is a first-line chemotherapeutic agent and is widely used in single-agent or combination therapy for advanced breast cancer and postoperative adjuvant chemotherapy [4]. Although TNBC is sensitive to chemotherapy, drug resistance to DOX has become a serious obstacle in current TNBC therapy [5,6,7,8], resulting patients enduring the toxic side effects and a poor prognosis.

Piperlongumine (PL) is a natural alkaloid that was first isolated from *Piper longum* L. in the 1960s [9]. It has multiple biological activities [10], and its anticancer activity has recently become a research hot spot. A previous study identified PL as a potent cytotoxic compound against various cancer cells with reliable selectivity. Its killing mechanism involves reactive oxygen species (ROS) accumulation in cells with a cancer genotype, regardless of p53 status [11], suggesting that PL could be a promising natural compound for cancer therapy. The role of PL in killing cancer cells includes inhibiting proliferation, inducing apoptosis [12,13], promoting ROS production [11,13], inhibiting migration and invasion [14,15], and sensitizing chemotherapy drugs [16,17,18], which activate multiple signaling pathways, including MAPK [13], PI3K-Akt-mTOR [12,19], nuclear factor kappa B (NF-κB) [20,21], GSTP1 [22], and TrxR1 [23]. At present, there are few studies on the role of PL in breast cancer, especially in TNBC. PL can induce TNBC apoptosis by inhibiting the PI3K-Akt-mTOR signaling pathway [12]. PL can be used as a direct inhibitor of STAT3 against TNBC in vitro and in vivo [24]. However, the role of PL in the entire JAK2-STAT3 pathway in TNBC, as well as the combined application of PL and DOX in any cancer, has not yet been reported. Based on these findings, we intend to apply PL to combination therapy, hoping to reduce the toxic side effects and improve the prognosis of DOX.

To find a solution to the above problems in the clinical treatment of TNBC, we investigated the anticancer effect of PL alone and in combination with DOX in two TNBC cell lines. We evaluated whether PL could enhance DOX by suppressing the JAK2-STAT3 pathway. Our results showed that the combination treatment of PL and DOX synergistically inhibited cell growth and induced apoptosis by suppressing Bcl-2 and p-STAT3 levels. We believe that this novel combination therapy could be a promising strategy for TNBC patients.

## 2. Results

### 2.1. Piperlongumine Inhibits the Proliferation of TNBC Cells

**Figure 1:**
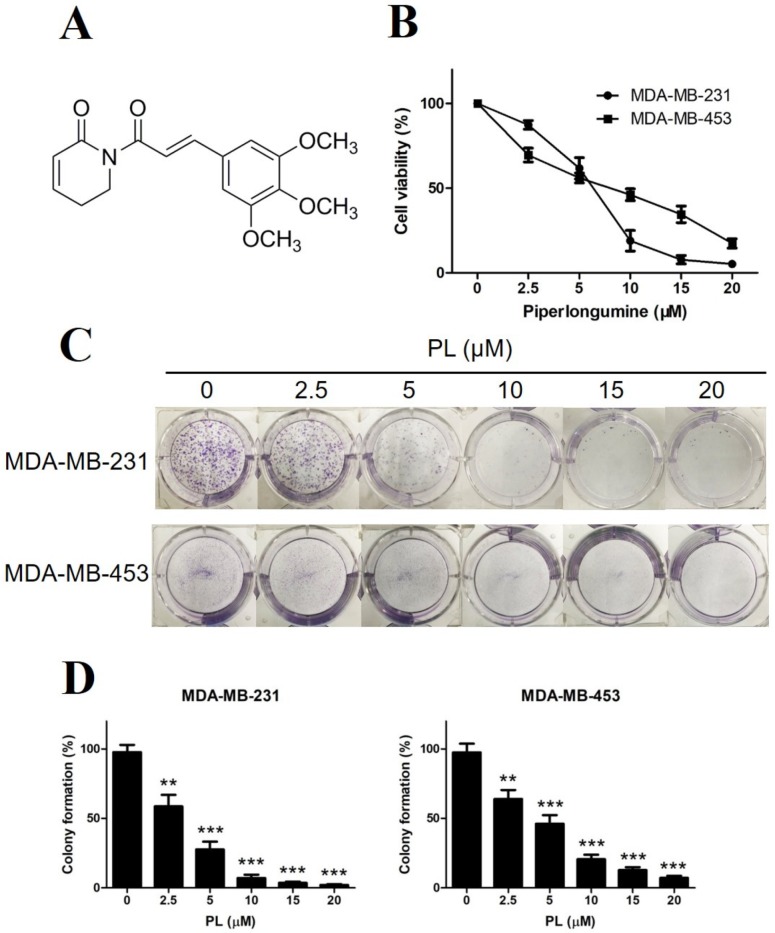
Piperlongumine inhibits proliferation of TNBC cells. (**A**) Molecular structure of piperlongumine. (**B**) MDA-MB-231 and MDA-MB-453 cells were treated with different concentrations of PL for 48 h, DMSO was used as a vehicle control, and cell viability was detected by methylthiazolyldiphenyl-tetrazolium bromide (MTT). (**C**) Both cells were seeded in 6-well plates for 24 h and then treated with various concentrations of PL for the next 10–14 days. (**D**) Colony formation rate was counted by ImageJ software. Colony formation rate = (number of colonies/number of seeded cells) × 100%. ** *p* < 0.01, *** *p* < 0.001 compared to the control.

Since PL has become a research hot spot in recent years as a natural small molecule with beneficial selective anticancer activity, we initially evaluated the inhibition of cell growth by PL in two human TNBC cell lines. MDA-MB-231 and MDA-MB-453 cells were exposed to various concentrations of PL for 48 h and were detected by cell viability assay. As shown in Figure 1B, PL inhibited the growth of both TNBC cell lines in a dose-dependent manner, and MDA-MB-231 cells appeared to be more sensitive than MDA-MB-453 cells. To evaluate the long-term inhibition effect of PL, we then performed colony formation assays.

Both MDA-MB-231 and MDA-MB-453 cells were treated with various concentrations of PL and continued to grow for 10–14 days. The results showed that PL also inhibited colony formation in a dose-dependent manner (Figure 1C,D), which was consistent with the cell viability assay.

### 2.2. Piperlongumine Induces Apoptosis in TNBC Cells

**Figure 2:**
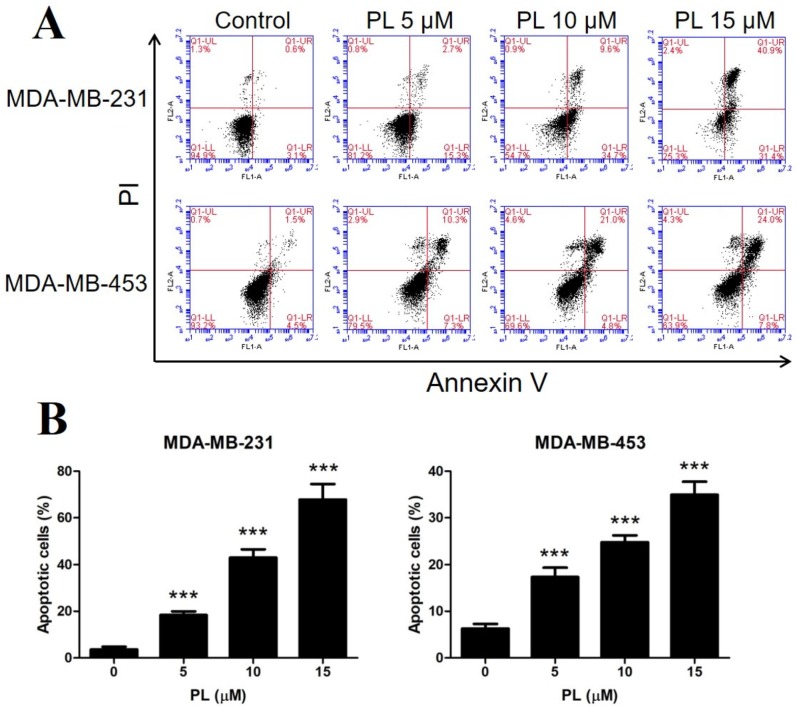
Piperlongumine induces apoptosis in TNBC cells. (**A**) MDA-MB-231 and MDA-MB-453 cells were treated with 5, 10, or 15 µM PL for 48 h and stained with Annexin V/PI. DMSO was used as a vehicle control. The apoptotic rate was then detected by flow cytometry. (**B**) Cells in the Annexin V-positive area were considered apoptotic. *** *p* < 0.001 compared to the control.

To uncover the mechanism of PL inhibiting TNBC proliferation, cell apoptosis analysis was performed. Both TNBC cell lines were treated with certain concentrations of PL, stained with Annexin V- fluorescein isothiocyanate (FITC)/propidium iodide (PI), and analyzed by flow cytometry. The results showed that PL induced apoptosis in both TNBC cell lines in a dose-dependent manner. PL at 15 µM significantly increased the apoptotic rate to over 70% in MDA-MB-231 cells and to over 30% in MDA-MB-453 cells (Figure 2A,B, respectively). MDA-MB-231 cells appeared to be more sensitive than MDA-MB-453 cells, which is consistent with our previous tests.

### 2.3. Piperlongumine Downregulates Bcl-2 and Inhibits the JAK2-STAT3 Pathway Activation in TNBC Cells

**Figure 3:**
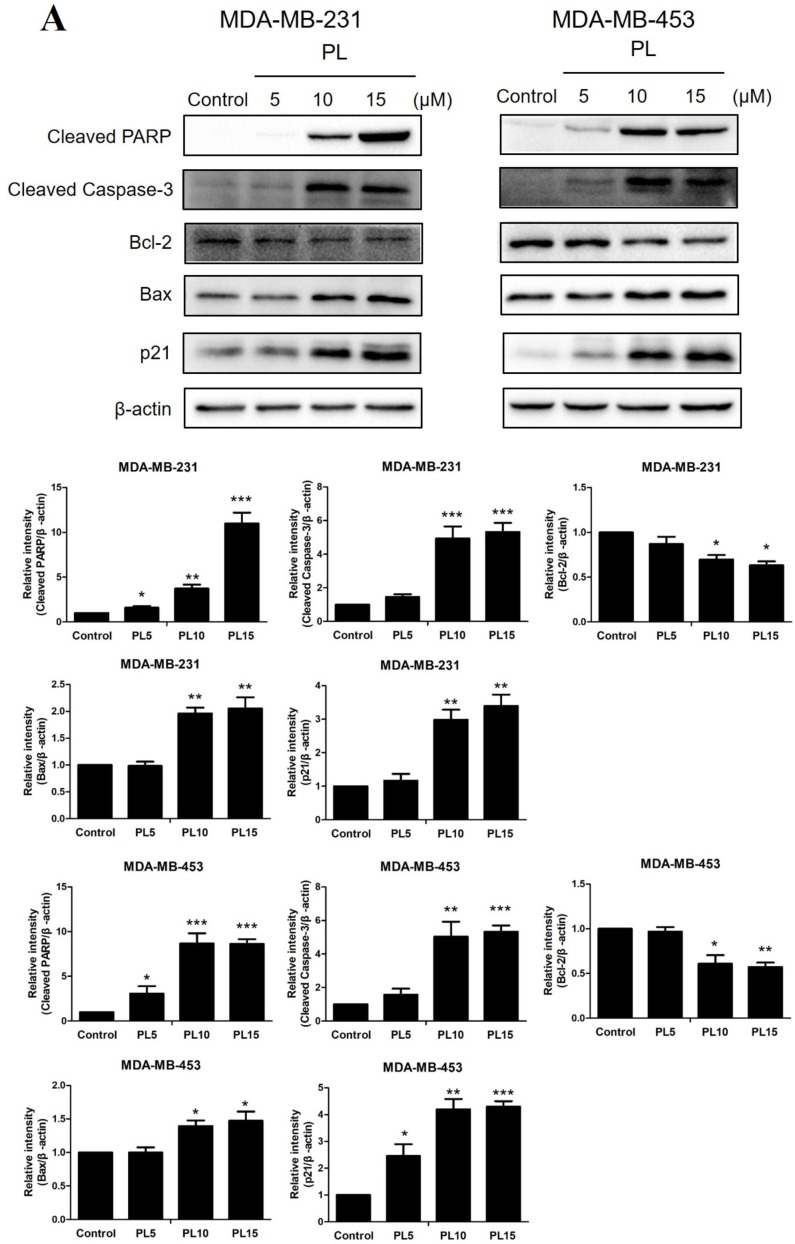

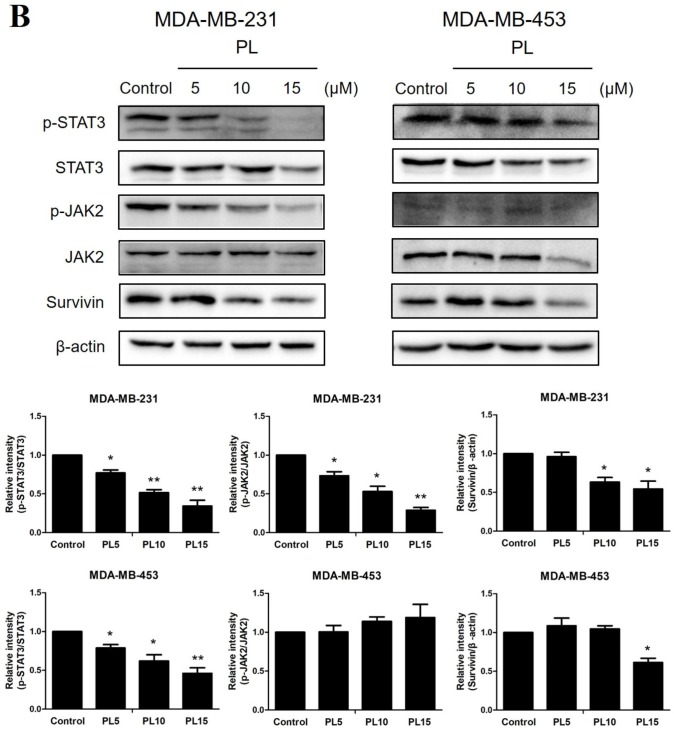
Piperlongumine downregulates Bcl-2 through inhibition of the JAK2-STAT3 pathway. (**A**,**B**) Both TNBC cell lines were treated with 5, 10, or 15 µM PL, or DMSO as a vehicle control for 24 h. Cell lysates were then subjected to Western blot analysis. Apoptosis-related proteins, including Bcl-2, Bax, cleaved PARP, cleaved caspase-3, and p21, were detected. p-STAT3, STAT3, p-JAK2, JAK2, and survivin levels were then measured. β-actin was used as an internal control. * *p* < 0.05, ** *p* < 0.01, *** *p* < 0.001 compared to the control.

We then investigated the effect of PL on the expression of apoptosis-related proteins by Western blot analysis in both TNBC cell lines. The results showed that PL significantly decreased the expression of Bcl-2 in a dose-dependent manner, while the upregulation of Bax was observed. Moreover, the expression of cleaved caspase-3 and cleaved PARP in the downstream apoptotic performers were increased significantly, confirming the proapoptotic effect of PL. In addition, the expression of the cell cycle arrest-associated protein p21 was also increased by PL (Figure 3A).

To investigate the underlying molecular mechanism of PL-induced TNBC apoptosis, we next examined the effects of PL on the JAK2-STAT3 pathway. Western blot analysis showed that PL decreased STAT3 phosphorylation in a dose-dependent manner in both TNBC cell lines, while the inhibition effect was stronger on MDA-MB-231 than MDA-MB-453. JAK2, the upstream protein of STAT3, was also decreased by PL in MDA-MB-231 cells but was not clearly observed in MDA-MB-453 cells, which may be due to its low expression. In addition, PL decreased survivin levels in both cell lines, indicating that PL could effectively resist the antiapoptotic effect of TNBC (Figure 3B). Taken together, these results demonstrated that PL could effectively induce apoptosis through inhibition of the JAK2-STAT3 pathway.

### 2.4. Piperlongumine and Doxorubicin Synergistically Inhibit the Proliferation of TNBC Cells

**Figure 4:**
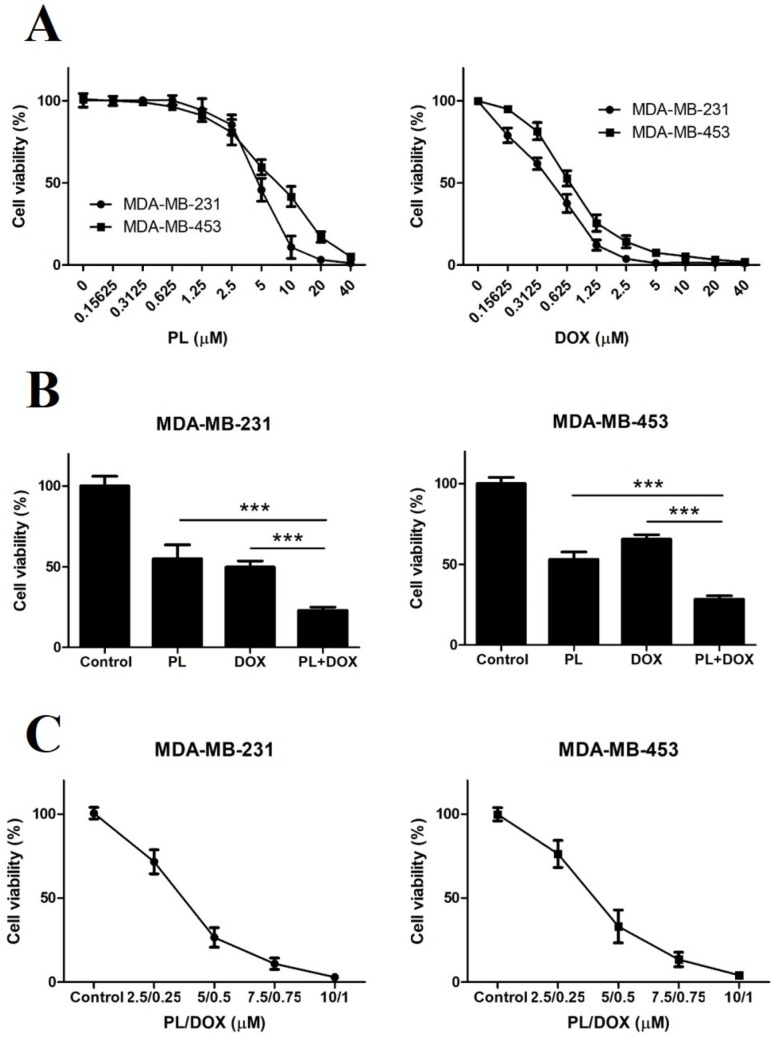
Piperlongumine and doxorubicin synergistically inhibit the proliferation of TNBC cells. (**A**) MDA-MB-231 and MDA-MB-453 cells were treated with various concentrations of PL or DOX for 72 h, and DMSO was used as a vehicle control. Cell viability was detected by MTT. (**B**) Both cells were treated with DMSO, 5 µM PL, 0.5 µM DOX, or in combination for 72 h. Cell viability was detected by MTT. The CI was calculated using CompuSyn. The CI values of the combination treatment were 0.57 in MDA-MB-231 cells and 0.61 in MDA-MB-453 cells. (**C**) Combination treatment of PL and DOX inhibited the growth of TNBC cells in a dose-dependent manner. Both TNBC cell lines were treated with the indicated concentrations of PL and DOX for 72 h, and cell viability was detected by MTT. *** *p* < 0.001 compared to the compared group.

To reduce the toxic side effects and improve the prognosis of DOX, we intended to study the synergistic anticancer effects of PL and DOX. We first evaluated the inhibition effect of the two drugs alone or in combination in both TNBC cell lines. The results showed that PL inhibited TNBC cell growth in a dose-dependent manner, the IC_50_ value of PL was 4.693 μM on MDA-MB-231 cells while 6.973 μM on MDA-MB-453 cells. And DOX also inhibited TNBC cell growth in a dose-dependent manner, the IC_50_ value of DOX was 0.4131 μM on MDA-MB-231 cells while 0.7059 μM on MDA-MB-453 cells (Figure 4A).

We then investigated whether PL could enhance the effectiveness of DOX against TNBC cells. The results showed that the growth of TNBC cells was significantly inhibited by the combination treatment of 5 μM PL and 0.5 μM DOX in comparison to either agent alone in both TNBC cell lines (Figure 4B). The combination index (CI) values of the combination treatment were 0.57 in MDA-MB-231 cells and 0.61 in MDA-MB-453 cells, both of which were much less than 1.0, indicating a synergistic effect. Besides, different ratios of combination treatment of PL and DOX inhibited the cell growth of both TNBC cells in a dose-dependent manner (Figure 4C).

### 2.5. Piperlongumine and Doxorubicin Synergistically Induce Apoptosis by Inhibiting p-STAT3 in TNBC Cells

**Figure 5:**
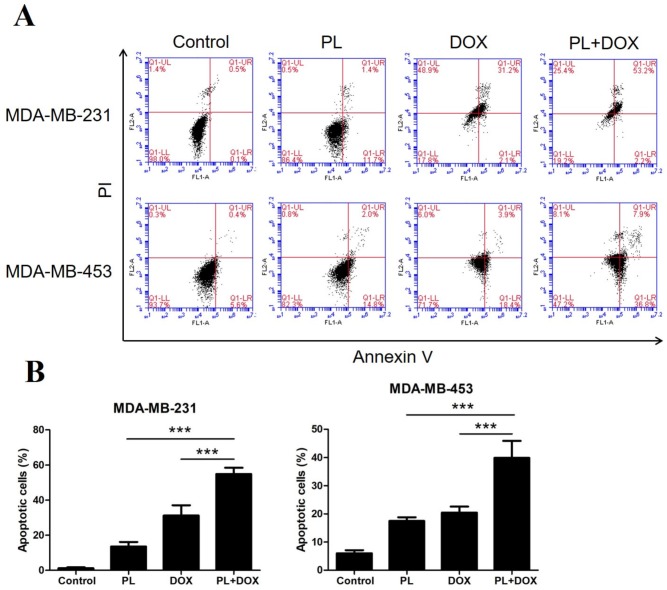

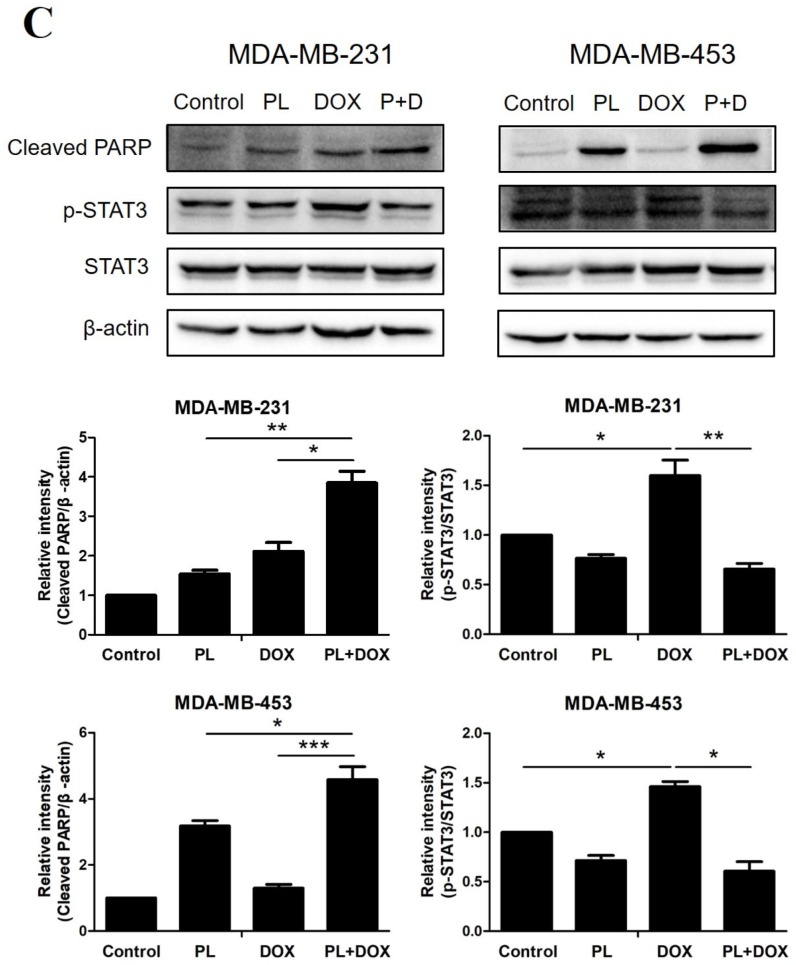
Piperlongumine and doxorubicin synergistically induce apoptosis and inhibit p-STAT3 in TNBC cells. (**A**) Both MDA-MB-231 and MDA-MB-453 cells were treated with DMSO, 5 µM PL, 0.5 µM DOX, or with a combination for 48 h. Cells were then harvested and stained with Annexin V/PI. The apoptotic rate was detected by flow cytometry. (**B**) Cells in the Annexin V-positive area were considered apoptotic. (**C**) Both TNBC cell lines were treated with DMSO, 5 µM PL, 0.5 µM DOX, or with a combination for 24 h. Cell lysates were then subjected to Western blot analysis. Cleaved PARP, p-STAT3, and STAT3 levels were measured. β-actin was used as an internal control. * *p* < 0.05, ** *p* < 0.01, *** *p* < 0.001 compared to the compared group.

Since PL and DOX could independently induce apoptosis in TNBC, we anticipated that the two agents may have a synergistic effect; so we performed apoptosis analysis with a combination treatment. MDA-MB-231 and MDA-MB-453 cells were treated with 5 μM PL and 0.5 μM DOX alone or in combination for 48 h. Cells were then stained with Annexin V-FITC/PI and analyzed by flow cytometry. As shown in Figure 5A,B, the combination of PL and DOX significantly induced apoptosis in both TNBC cell lines and showed a synergistic effect compared with either agent alone.

Combination effects on cleaved PARP and p-STAT3 levels were also detected by Western blot analysis. Compared with treatment alone, the combination of PL and DOX significantly increased the cleaved PARP level (Figure 5C). The results showed that PL reduced the expression of p-STAT3, while DOX showed the opposite effect. However, the upregulation effect of DOX on p-STAT3 was reversed when the two agents were treated in combination, indicating that PL may effectively improve drug resistance to DOX.

### 2.6. Piperlongumine and Doxorubicin Synergistically Suppress Xenograft Tumor Growth of TNBC Cells

**Figure 6:**
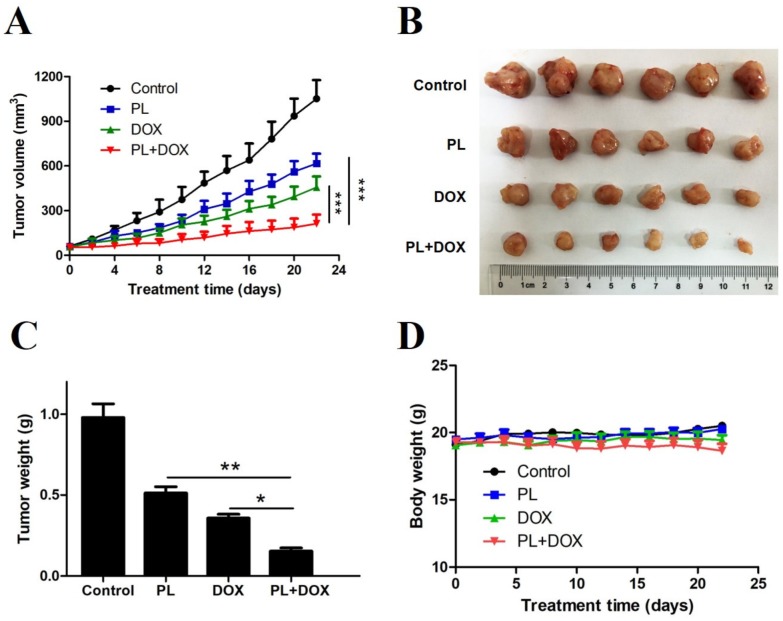
Piperlongumine and doxorubicin synergistically suppress xenograft tumor growth of TNBC cells. (**A**) Female nude mice were injected with 5 × 10^6^ MDA-MB-231 cells in the right mammary gland and were randomly divided into 4 groups (n = 6 per group) when the tumor volume reached 50 mm^3^. Nude mice were then treated with DMSO, PL, DOX, or with a combination via i.p. injections every other day. (**B**) All nude mice were sacrificed on day 22 after treatment, and tumors were collected and recorded. (**C**) Each tumor collected from nude mice was weighed and analyzed. (**D**) Treatment with PL and DOX or a combination did not show a significant effect on mouse body weight. * *p* < 0.05, ** *p* < 0.01, *** *p* < 0.001 compared to the compared group.

To assess the synergistic effects of PL and DOX in vivo, we treated 24 female nude mice bearing MDA-MB-231 tumor xenografts with intraperitoneal (i.p.) injections of vehicle, 4 mg/kg PL, 0.8 mg/kg DOX, or with a combination for 22 days. PL or DOX alone significantly decreased the tumor growth rates. Notably, the combination of PL and DOX synergistically suppressed in vivo tumor growth (Figure 6A,B). The tumor weight was consistent with tumor growth, and the tumor weight of the combination group was significantly lower than that of the agent alone group (Figure 6C). Changes in body weights were not significantly different between the vehicle control and other groups (*p* > 0.5) (Figure 6D), indicating no significant toxicity occurred in all treatment groups.

## 3. Discussion

Current clinical treatment of TNBC relies mainly on chemotherapy due to a lack of targeted drugs, but its toxic side effects and poor prognosis have not been well solved. To improve this dilemma, we focused on PL, a natural alkaloid originally isolated from pepper plants. Recent research has reported that PL selectively kills cancer cells but not normal cells [11], which makes it a promising anticancer agent.

The killing effect of PL has been confirmed in various cancer cells, and PL can synergize with cisplatin against head and neck cancer [16], with paclitaxel against ovarian cancer [25], and with gemcitabine against pancreatic cancer [17]. Research on the combined application of PL and DOX in breast cancer has not yet been reported. In the present study, we identified PL potently inhibiting growth and inducing apoptosis of TNBC cells in vitro and in vivo, which is consistent with previous reports [12,24]. Then, we conducted a combination study and demonstrated that PL and DOX synergistically inhibited proliferation (Figure 4), induced apoptosis (Figure 5), and suppressed tumor growth in a nude mice model (Figure 6). Western blot results showed that the expression of cleaved PARP significantly increased (Figure 5) when PL and DOX were combined, while the expression of p-STAT3 significantly decreased (Figure 5), indicating that this combination can effectively increase apoptosis by inhibiting the JAK2-STAT3 pathway in TNBC.

Apoptosis is a strictly controlled pattern of programmed cell death that is essential for the normal growth and the development of multicellular organisms. Cancer cells can proliferate indefinitely because they break through the limitations of apoptosis. The Bcl-2 family is the most important protein in apoptosis research and is divided into two categories: antiapoptotic proteins, such as Bcl-2, and proapoptotic proteins, such as Bax. Both proteins play a role in apoptotic regulation [26]. Proapoptotic drugs cleave apoptosis executioners, such as caspase 3 and its substrate PARP, by regulating the expression of Bcl-2/Bax [27,28]. In the present study, we observed a decrease in Bcl-2 expression, and increased expression in Bax, cleaved caspase-3 and cleaved PARP, indicating PL showed a strong ability to induce apoptosis in both TNBC cell lines (Figure 3). We also found that PL upregulated p21, which is associated with tumor differentiation, proliferation, invasion, and metastasis. Therefore, upregulation of p21 indicated that PL may improve the prognosis of TNBC. DOX is known to induce apoptosis in breast cancer cells by reducing Bcl-2 expression as a traditional chemotherapy drug [29]. Thus, we wondered whether the combination of two drugs would have a synergistic proapoptotic effect. Flow cytometry data showed that the apoptotic rate was significantly higher when two agents were combined. Western blot data showed upregulation of cleaved PARP and downregulation of p-STAT3 when the agents were combined, which confirmed our hypothesis and explained the mechanism of this synergy effect.

The JAK2-STAT3 signaling pathway plays a crucial role in the proliferation, survival, and drug resistance of breast cancer [30,31]. At present, some specific small molecule inhibitors targeting STAT3 have been developed, but none of them have entered the clinical stage. Therefore, finding novel drugs targeting STAT3 activation remains an important scientific and clinical challenge. Previous research reported that DOX could activate p-STAT3, which may be responsible for drug resistance during TNBC clinical treatment [32]. Our data confirmed that DOX significantly enhances STAT3 phosphorylation in both TNBC cell lines, with no significant change in total STAT3 expression. This could be the evidence to why DOX causes poor prognosis in TNBC therapy. PL killed a panel of breast cell lines as a direct inhibitor of STAT3 [24], and here we selected two TNBC cells with different levels of p-STAT3 as research models. Results indicated that PL showed a killing effect on both TNBC cells, while a more sensitive effect was observed in MDA-MB-231 than MDA-MB-453, where higher p-STAT3 expression in MDA-MB-231 could be one of the reasons. Inhibiting STAT3 activation is related to the sensitization of DOX toxicity as well as other chemotherapy drugs [33,34,35]. Here, we demonstrated that PL was able to downregulate p-STAT3, as well as its upstream and downstream genes, p-JAK2 and survivin. Our findings were consistent with previous data, indicating that PL could be used as a potent inhibitor of the JAK2-STAT3 pathway. Furthermore, we found that PL could reverse DOX-mediated STAT3 activation under combination treatment. Western blot results showed that a combination treatment significantly decreased STAT3 phosphorylation compared with DOX, indicating that PL may increase anticancer activity as well as prevent DOX resistance in a combination therapy of TNBC.

Our study reported for the first time that PL could synergize with DOX to inhibit cell growth and induce apoptosis in TNBC cells. Data demonstrated that the suppression of the JAK2-STAT3 pathway could be a novel mechanism of PL alone or in combination with DOX against TNBC. These results suggested that the combination of PL and DOX could be a promising strategy to reduce the toxic side effects and improve the prognosis in TNBC therapy.

## 4. Materials and Methods

### 4.1. Reagents

Piperlongumine and doxorubicin were purchased from ApexBio (Houston, TX, USA). Both agents were dissolved in dimethyl sulfoxide (DMSO) and diluted 1:1000 in culture medium.

### 4.2. Cell Culture

Triple-negative breast cancer (TNBC) cell lines MDA-MB-231 and MDA-MB-453 were purchased from American Type Culture Collection (ATCC, Manassas, VA, USA). Both cells were cultured in Dulbecco’s Modified Eagle’s Medium (DMEM, Thermo Fisher Scientific, Waltham, MA, USA), supplemented with 10% FBS (Thermo Fisher Scientific, Waltham, MA, USA) and 100 U/mL penicillin and streptomycin (Thermo Fisher Scientific, Waltham, MA, USA). Both cells were cultured with 5% CO_2_ in a 37 °C incubator.

### 4.3. Cell Viability Assay

Both cells were seeded at (3–10) × 10^3^ cells/well in 96-well plates with overnight incubation and exposed to certain concentrations of PL and DOX, alone or in combination, for 48 h of incubation. Then an MTT working solution was added and absorbance in each well was measured at 570 nm using an Epoch microplate reader (BioTek, Winooski, VT, USA). The combination index (CI) values were calculated using CompuSyn software (Version 1.0, ComboSyn, Inc., New York, NY, USA). A CI < 1.0 can be considered as signifying synergy.

### 4.4. Cell Apoptosis Analysis

Both cells were seeded in 6-well plates for overnight incubation, and then treated with PL and DOX for 48 h. Cells were then harvested, washed twice with ice-cold PBS, and double-stained with FITC-conjugated Annexin V and PI in binding buffer for 15 min. The rate of apoptosis was evaluated using Accuri C6 flow cytometer (BD, Franklin Lakes, NJ, USA).

### 4.5. Western Blot Analysis

Cells and tumor tissues were homogenized in protein lysate buffer and collected after centrifugation at 12,000× g for 10 min at 4 °C. The protein concentrations were determined by BCA protein assay kit (Beyotime, Shanghai, China). Then total proteins with equal amounts were subjected to sodium dodecyl sulfate-polyacrylamide gel electrophoresis (SDS-PAGE) and electro-transferred onto poly-vinylidene difluoride (PVDF) membranes. The blots were blocked for 1 h at room temperature with 5% nonfat milk and then incubated with specific primary and secondary antibodies. The β-actin (Beyotime, Shanghai, China) was used as an internal control. The primary antibodies included Bcl-2, Bax, cleaved caspase3, cleaved PARP, p21, p-JAK2, JAK2, p-STAT3, STAT3, and survivin (Cell Signaling Technology, Danvers, MA, USA). 

### 4.6. Colony Formation Assay

Both cells were seeded at 300 cells/well or 1000 cells/well in 6-well plates for overnight incubation and treated with certain doses of PL. After incubation for a further 10–14 days, cells were then fixed with 4% formalin and stained with 0.1% crystal violet. The number of colonies in each well was counted by ImageJ software (Version 1.49, National Institutes of Health, Bethesda, MD, USA). 

### 4.7. In Vivo Antitumor Study

All animal study procedures were performed in accordance with protocols approved by the Institutional Animal Care and Use Committee of Xi’an Medical University. Five-week-old female nude mice were purchased from HuaFuKang Bioscience (Beijing, China) and were housed in an animal barrier facility of Xi’an Medical University. MDA-MB-231 cells (5 × 10^6^) were injected subcutaneously into the right mammary gland. When tumors volume reached 50 mm^3^, mice were randomized into four treatment groups and were treated by intraperitoneal (i.p.) injection of the vehicle, or 4 mg/kg PL, or 0.8 mg/kg DOX, or with a combination of PL and DOX every other day. Tumors were measured every other day using a caliper and volumes were calculated as (length × width^2^)/2. At the end of treatment, nude mice were sacrificed and tumors were removed for Western blot analysis.

### 4.8. Statistical Analysis

The results of all tests, except for in vivo antitumor study, expressed as the mean ± SD of at least three independent experiments. All statistical analysis were conducted using GraphPad Prism software (Version 5.0, GraphPad Software Inc., La Jolla, CA, USA). Statistical significance between multiple treatment groups was analyzed using one-way ANOVA. *p* < 0.05 is considered statistically significant. The combination index (CI) values were calculated using CompuSyn software. CI < 1.0 is considered to be synergistic.

## Highlights

PL induces apoptosis of TNBC cells by downregulation of Bcl-2 and inhibition of p-STAT3. PL and DOX synergistically inhibit the proliferation of TNBC cells. PL and DOX synergistically induce apoptosis by inhibiting p-STAT3 in TNBC cells. PL and DOX synergistically suppress TNBC tumor growth in vivo.
